# Deep Neural Network Models for Predicting Chemically Induced Liver Toxicity Endpoints From Transcriptomic Responses

**DOI:** 10.3389/fphar.2019.00042

**Published:** 2019-02-05

**Authors:** Hao Wang, Ruifeng Liu, Patric Schyman, Anders Wallqvist

**Affiliations:** ^1^The Henry M. Jackson Foundation for the Advancement of Military Medicine, Inc., Bethesda, MD, United States; ^2^Department of Defense Biotechnology High Performance Computing Software Applications Institute, Telemedicine and Advanced Technology Research Center, United States Army Medical Research and Materiel Command, Frederick, MD, United States

**Keywords:** machine leaning, classification model, toxicity prediction, artificial neural network, biliary hyperplasia, liver fibrosis, liver necrosis

## Abstract

Improving the accuracy of toxicity prediction models for liver injuries is a key element in evaluating the safety of drugs and chemicals. Mechanism-based information derived from expression (transcriptomic) data, in combination with machine-learning methods, promises to improve the accuracy and robustness of current toxicity prediction models. Deep neural networks (DNNs) have the advantage of automatically assembling the relevant features from a large number of input features. This makes them especially suitable for modeling transcriptomic data, which typically contain thousands of features. Here, we gaged gene- and pathway-level feature selection schemes using single- and multi-task DNN approaches in predicting chemically induced liver injuries (biliary hyperplasia, fibrosis, and necrosis) from whole-genome DNA microarray data. The single-task DNN models showed high predictive accuracy and endpoint specificity, with Matthews correlation coefficients for the three endpoints on 10-fold cross validation ranging from 0.56 to 0.89, with an average of 0.74 in the best feature sets. The DNN models outperformed Random Forest models in cross validation and showed better performance than Support Vector Machine models when tested in the external validation datasets. In the cross validation studies, the effect of the feature selection scheme was negligible among the studied feature sets. Further evaluation of the models on their ability to predict the injury phenotype *per se* for non-chemically induced injuries revealed the robust performance of the DNN models across these additional external testing datasets. Thus, the DNN models learned features specific to the injury phenotype contained in the gene expression data.

## Introduction

Toxicity prediction is a key element in evaluating the safety of drugs and chemicals (Raies and Bajic, 2016). Currently, the standard methods of toxicity evaluation are based on animal experiments to identify mechanisms of action and potential toxic effects (Benigni, 2016; Raies and Bajic, 2016). Recent advances in biological and computational modeling techniques are accelerating the development of a large number of animal-free assays and *in silico* models for toxicity testing (Blomme and Will, 2016). However, extensive work on these assays and models is still needed if they are to adequately address the central challenge of providing accurate prediction of toxicity endpoints and thus become valid replacements for traditional methods (Benigni, 2016).

Gene expression (transcriptomic) data have been widely used since the introduction of microarrays to elucidate the links between chemical exposures and the adverse effects they induce (Brockmeier et al., 2017). In particular, transcriptomic data can be used to discover genes and pathways associated with adverse effects and provide mechanistic insights, using multiple computational methods (Brockmeier et al., 2017), including those that involve machine learning (Thomas et al., 2001; Hamadeh et al., 2002; Steiner et al., 2004; Huang et al., 2008; Kohonen et al., 2017). Increasingly, research is focused on using gene expression signatures as predictors of a chemical’s toxicological class (Zidek et al., 2007; Low et al., 2011; Kim and Shin, 2014; AbdulHameed et al., 2016). A recent work by Su et al. (2018) suggests that careful feature engineering could achieve high accuracy in predictions of liver injury using transcriptomic data. An advantage of the transcriptomic approach is that gene expression data may provide an early indication of toxicity, given that toxicant-induced changes in gene expression are often detectable before chemical, histopathological, or clinical observations (Ulrich and Friend, 2002). These studies have typically employed traditional supervised or unsupervised machine-learning methods, such as Support Vector Machines (SVMs) or Random Forests (RFs).

Recently, Deep Neural Networks (DNNs) (Hinton et al., 2006) have achieved notable success in many domains of machine-learning applications, including the biomedical sciences (Schmidhuber, 2015; Webb, 2018). In the context of toxicity prediction, DNN models have been assessed in several comprehensive studies for building quantitative structure–activity relationship models of different absorption, distribution, metabolism, excretion, and toxicity (ADMET)-properties based on chemical structural features (Ma et al., 2015; Xu et al., 2015; Goh et al., 2017). Aliper and colleagues developed both DNNs and SVMs based on *in vitro* transcriptomic data generated by the Connectivity Map (CMap) at the Broad Institute (Subramanian et al., 2017) to classify therapeutic categories for chemicals, and found DNNs to have superior classification performance (Aliper et al., 2016).

Transcriptomes contain thousands to tens of thousands of input variables that can be used as features in machine learning. These range from the most granular, of individual features representing the expression levels of single genes, to more focused sets or combinations of genes or pathways. The performance of traditional machine-learning methods typically depends critically on manually selecting and tuning these features to find the appropriate transcriptomic feature sets for model construction (Steiner et al., 2004; Huang et al., 2008). A key advantage of DNNs is their capability of discovering the representations that are relevant to solving a classification problem from the input features automatically, with little if any manual intervention (LeCun et al., 2015). In principle, DNNs should be able to handle large numbers of transcriptomic features, provided that ample training data are available. In practice, however, this advantage is diminished by the lack of training data, and some consideration is still required to construct or select the appropriate input variables.

Here, we used whole-genome DNA microarray data to construct DNN models of three histopathological endpoints (biliary hyperplasia, fibrosis, and necrosis), based on liver toxicity studies available in the Open Toxicogenomics Project-Genomics Assisted Toxicity Evaluation System (TG-GATEs) (Igarashi et al., 2015) and DrugMatrix (Ganter et al., 2006), and evaluated their performance on multiple, independent testing datasets. Because the liver is the major site where drugs and exogenous toxins are metabolized, these extensive dataset compilations make DNN modeling feasible for complex *in vivo* liver disease phenotypes induced by chemical exposures. To gage the performance levels of our models and evaluate the influence of feature selection, we built both single- and multi-task DNNs for the three liver toxicity endpoints, compared the performance of DNNs to that of SVMs and RFs, and examined the robustness of the models in predicting the liver injury phenotypes in experimental datasets derived from non-chemically induced injuries. We further investigated the impact of feature selection strategy on prediction accuracy, by building models using multiple sets of gene- and pathway-level features. Overall, our results suggest that DNNs offer a practical and robust modeling strategy to predict chemically induced liver injury from transcriptomic data.

## Materials and Methods

### Transcriptomic Data

We developed machine-learning models for three commonly evaluated histopathology endpoints liver endpoints biliary hyperplasia, liver fibrosis, and liver necrosis using data publicly available from two large-scale toxicogenomics databases, DrugMatrix (Ganter et al., 2006)^1^ and Open TG-GATEs (Igarashi et al., 2015)^2^. These databases contain data that match chemical exposures with transcriptomic changes in multiple tissues of Sprague–Dawley rats to graded histopathology assessments. We downloaded the rat *in vivo* liver microarray datasets based on the Affymetrix GeneChip Rat Genome 230 2.0 Array from TG-GATEs and DrugMatrix. The raw dataset contains whole genome microarray expression data for liver and kidney from 6,765 and 2,218 rats, respectively. According to our previous protocol (Tawa et al., 2014; Te et al., 2016), we assessed the quality of the arrays and removed outlier arrays and renormalized the remaining data. For both datasets, we further removed samples where histopathology scores for the three studied endpoints were missing. For some chemicals, all of the exposure conditions, i.e., chemical-time-dose combinations, did not induce any of the three endpoints, and we removed samples related to these chemicals to mitigate the problem of data imbalance. The final training data (Table 1) included all samples of all available exposure conditions. We built and evaluated the models using 10-fold cross validation. The detailed information of used samples, chemical exposure conditions, and histopathology outcomes are given in Supplementary Table S1.

**Table 1:** Summary of training and testing data used in this study.

Source	Number of samples						Reference
		
	Biliary Hyperplasia		Liver Fibrosis		Liver Necrosis		
				
	+	-	+	-	+	-	
**Training data**							
Open TG-GATEs	91	2,233	37	2,287	275	2,049	Igarashi et al., 2015
DrugMatrix DB	38	661	27	672	179	520	Ganter et al., 2006
**External testing data**							
Gene Expression Omnibus	20	52	19	53	30	42	Ippolito et al., 2016
	N/A	N/A	N/A	N/A	2	15	Stallings et al., 2014
	0	269	0	269	0	269	Eun et al., 2015
	N/A	N/A	3	32	N/A	N/A	Brown et al., 2016


Our models were evaluated on independent external datasets. The first dataset was obtained from rats repeatedly exposed to four chemicals at multiple doses and time points [(Ippolito et al., 2016); Gene Expression Omnibus accession number, GSE70559] exhibiting liver injury endpoints contained in the training data. The four chemicals are part of the TG-GATEs and DrugMatrix dataset, but with different doses and exposure durations. In order to assess endpoint predictions using this data, we first removed the training samples in TG-GATEs and DrugMatrix related to the four chemicals and built DNN and SVM models on the remaining training data.

To further assess the ability of the models to predict liver-injury phenotypes, we constructed five additional independent testing sets from publically accessible rat liver *in vivo* data on (1) liver necrosis caused by heat stress [(Stallings et al., 2014); GEO accession number GSE56740], (2) three endpoints for bile duct ligation [(Sutherland et al., 2018); GEO accession number GSE87696], (3) liver fibrosis after exposure to nevirapine, galactosamine, and their combination [(Brown et al., 2016); GEO accession number GSE72076], (4) exposure to five chemicals that had no impact on liver histopathology [(Eun et al., 2015); GEO accession number GSE49631], and (5) biliary hyperplasia and liver necrosis after exposure to methapyrilene [(Slopianka et al., 2017); GEO accession number GSE95470].

The first two datasets involved the use of non-chemical treatments, i.e., heat shock (Stallings et al., 2014) and bile duct ligation (Sutherland et al., 2018). The third was obtained using two chemicals, nevirapine and galactosamine (Brown et al., 2016), of which only galactosamine was present in the training data. The fourth involved five chemicals, i.e., pyrazinamide, ranitidine, enalapril, carbamazepine, and chlorpromazine (Eun et al., 2015), of which only carbamazepine was present in the training data. The final dataset, involved the use of methapyrilene at a dose and exposure duration different from those used in the training data (Slopianka et al., 2017). In short, these datasets thus represented physiological and chemical perturbations independent of the training data.

Four external datasets (Ippolito et al., Stallings et al., Brown et al., and Eun et al.) had sample-level histopathology annotations, i.e., the transcriptome of each sample was directly linked to its histopathological injury score. Therefore, standard contingency tables allowed us to evaluate the models based on these data. In contrast, Sutherland et al. and Slopianka et al. data did not include sample-level histopathology annotations, although they did include mean injury scores at multiple time points and/or doses (exposure conditions). These data allowed us to investigate the correlation and consistency between experimental results and model predictions. Table 1 summarizes all training datasets and four external testing datasets. The detailed information of samples, chemical exposure conditions, and histopathology outcomes of these external datasets are given in Supplementary Tables S1, S2.

### Feature Selection

To investigate the impact of varying the input features on the performance of our model, we constructed gene- and pathway/co-expression module-level feature sets, each of which represented a specific way of extracting liver toxicity information from the transcriptome. Table 2 shows the 13 constructed datasets that we used as the model inputs.

**Table 2:** Summary of feature sets used in this study.

Name	Content	Reference
**Gene-level feature sets**		
*PTGS (all)*	1,331 genes	Kohonen et al., 2017
*PTGS (core)*	199 genes	Kohonen et al., 2017
*L1000*	978 genes	Subramanian et al., 2017
*Toxicity Module Gene*	1,312 genes belonging to 89 gene co-expression modules for chemically induced liver injury	Tawa et al., 2014; Te et al., 2016
*Toxicity Module (L1000)*	154 genes common to *Toxicity Module* and *L1000*	
*A200*	200 genes arbitrarily selected from the genome	
*A600*	600 genes arbitrarily selected from the genome	
*A1200*	1,200 genes arbitrarily selected from the genome	
**Pathway-/module-level feature sets**		
*MSigDB (C2)*	1,329 pathways in C2 collection	
*MSigDB (hallmark)*	50 pathways in Hallmark collection	Liberzon et al., 2015
*Toxicity Module*	89 gene co-expression modules for chemically induced liver injuries	Tawa et al., 2014; Te et al., 2016
*MSigDB (C2) L1000*	1,220 *MSigDB C2* pathways in which only genes in *L1000* are retained	
**Y-shuffled feature sets**		
*L1000 Y-Shuffle*	*L1000* data with random permutations of injury annotations	
*Toxicity Module Gene Y-Shuffle*	*Toxicity Module Gene* data with random permutations of injury annotations	


For gene-level feature sets, we defined a feature by the fold-change value calculated from the difference between the mean log-transformed gene expression values for samples in the treatment and control cohorts. Feature sets were chosen arbitrarily or according to previous data-mining results. We generated eight gene-level feature sets. The first three were based on data-mining techniques, two were formed by reducing the number of genes in these sets, and the remaining three were created by arbitrarily (randomly) selecting genes so that the number of genes was similar to that of the first five feature sets. The first three sets comprised the (1) predictive toxicogenomics space [*PTGS (all)*], composed of 1,331 genes considered related to cytopathology and drug-induced liver injury (Kohonen et al., 2017), (2) *L1000*, composed of 978 genes and considered as an adequate reduced representation of the whole-genome expression profile (Subramanian et al., 2017), and (3) *Toxicity Module Gene*, a set composed of genes belonging to 89 co-expression modules we previously identified as being associated with chemically induced liver injuries (Tawa et al., 2014; Te et al., 2016). To investigate the effect of varying the number of features on model performance, we generated two additional feature sets based on the first three: (4) *PTGS (core)*, a subset of *PTGS (all)* containing the 199 genes occurring in all 14 overlapping components (gene sets) of the latter (Kohonen et al., 2017), and (5) *Toxicity Module (L1000)*, composed of genes occurring in both *Toxicity Module* and *L1000*. Finally, to evaluate the impact of knowledge-based gene selection on the ability of predicting liver injury, we generated three non-curated gene sets: (6) *A200*, (7) *A600*, and (8) *A1200*, containing 200, 600, and 1,200 randomly selected genes, respectively. The number of genes in these sets spanned a range similar to that of the first five datasets.

For our pathway/module-level feature sets, we defined a feature as the expression score of an entire pathway/module (Schyman et al., 2018). Briefly, we first calculated the fold-change values for all genes occurring in the pathway/module set. Subsequently, we calculated the absolute value of each gene’s log-transformed fold-change value, as well as its average (μ_0_) and standard deviation (σ) across all genes. For a gene set (pathway), we calculated the average score ($\bar{X}$) of the absolute values. We estimated the significance of a gene set by its *p*-value, i.e., the probability of having a score ($\bar{X}$) more extreme than the calculated value. According to the Central Limit Theorem, the probability distribution for an average value is approximately normal with parameters μ_0_ and $\sigma /\sqrt{n}$, where *n* is the number of genes in the gene set. The *p*-value can be calculated as the upper tail of the *N* (μ_0_, $\sigma /\sqrt{n}$) distribution. The z-score, which is defined as

(1) $$z=\frac{\left(\bar{X}-\mu_{0}\right)}{\sigma /\sqrt{n}},$$

has a normal probability distribution, *N* (0, 1). We used this score as the expression score of the individual pathway/module.

We used the following four pathway and module feature sets: (1) the Molecular Signatures Database C2 collection [*MSigDB (C2)*], downloaded from the Molecular Signatures Database^3^ and containing 1,329 curated gene sets, including canonical pathways and gene sets representing expression signatures of genetic and chemical perturbations, (2) *MSigDB (hallmark)*, a collection of 50 gene sets generated by computationally mining all of the MSigDB data to reduce noise and redundancy, and considered to represent specific well-defined biological states or processes that display a coherent gene expression pattern (Liberzon et al., 2015), (3) *Toxicity Module*, a set of 89 co-expression modules associated with chemically induced liver injuries (Tawa et al., 2014; Te et al., 2016), and (4) *MSigDB (C2) L1000*, a set containing only *MSigDB (C2)* pathways in which at least one gene also occurred in *L1000*, and constructed to investigate how gene reductions in pathways affect model performance.

For each feature set, we calculated expression scores for samples in the training sets to obtain the input data matrices. Given an endpoint, histopathology annotations of samples provided the output vectors. The output vector represented the occurrence of the liver histopathology endpoint in the samples: 1 if the endpoint was found in the sample; and 0 otherwise.

The eight gene- and four pathway/module-level feature sets described above constituted the 12 training sets. To test for over-fitting of the model, we generated additional pathway-level datasets, *Toxicity Module Gene Y-Shuffle* and *L1000 Y-Shuffle*, by retaining the input matrix *of Toxicity Module Gene* and *L1000* while randomly shuffling the values in the output vectors for the training set (Table 2). Thus, we obtained 14 training sets for each histopathology endpoint associated with liver injury.

### Machine-Learning Classification Models

#### Data Imbalance

All datasets showed strong class imbalance, i.e., negative samples greatly outnumbered positive samples (Table 1). Classifiers may be biased towards major classes and, hence, show poor classification performance for minor classes (Lemaitre et al., 2017). To address this problem, we applied the Synthetic Minority Over-sampling Technique (SMOTE) (Chawla et al., 2002) to process the training data, and used multiple metrics, such as F1 and the Matthews correlation coefficient (MCC), to evaluate model performance. The MCC and F1 can be calculated from a confusion matrix as follows:

(2) $$F1=\frac{2}{\frac{1}{recall}+\frac{1}{precision}}=\;\frac{2}{\frac{TP}{TP+FN}+\frac{TP}{TP+FP}},$$

(3) $$\mathrm{MCC}=\frac{\mathrm{TP}\times \mathrm{TN}-\mathrm{FP}\times \mathrm{FN}}{\sqrt{\left(\mathrm{TP}+\mathrm{FP})(\mathrm{TP}+\mathrm{FN})(\mathrm{TN}+\mathrm{FP}\right)}(\mathrm{TN}+\mathrm{FN})},$$

where TP, TN, FP, and FN refer to the number of true positives, true negatives, false positives, and false negatives, respectively.

#### Deep Neural Networks

**FIGURE 1:**
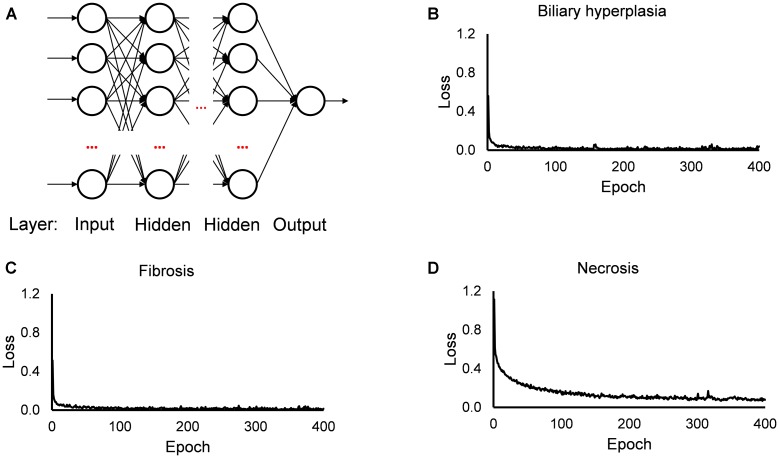
Diagram of single-task DNN and loss function during training. **(A)** Diagram of single-task DNN model. **(B–D)** Change of loss function during training single-task DNN model for Biliary hyperplasia, Fibrosis, and Necrosis, respectively.

The single-task DNNs used in this study were standard, fully connected multilayer perceptrons with a single neuron in the output layer. For each toxicity endpoint, we built 13 single-task DNNs based on the 13 feature sets. For the DNN calculations, we used the open source Python library Keras^4^ on top of TensorFlow^5^ as the backend, a ReLU activation function for the hidden layers, a sigmoid activation function for the output layer, the Adam optimizer, a binary cross-entropy loss function, a kernel initializer with a normal distribution, early stopping and a dropout technique for all input and hidden layers. For each single-task DNN, we optimized the hyperparameters (i.e., the number of hidden layers, number of nodes in the layers, batch size, and dropout rate; Supplementary Table S3) by a grid search technique with cross validation, using the F1 score as the objective metric. Figure 1 shows the diagram of our single-task neural network, and the values of training loss. In all investigated cases, the loss values became flat within 200 epochs.

Multi-task learning try to solve the classification of three endpoints at the same time. To test the performance of multi-task learning strategy in predicting liver injury, we constructed multi-task DNNs with hard parameter sharing (Caruana, 1997). For our multi-task model, the three endpoints shared the first several hidden layers, while each endpoint retained task-specific layers to generate the relevant liver injury outputs. The DNN calculations were performed as described for single-task DNNs. We built the multi-task DNNs and optimized the hyperparameters (i.e., the number of shared and task-specific hidden layers, number of nodes in the hidden layers, relative weighting between each task’s loss, and dropout rate; Supplementary Table S3) by a grid search technique with cross validation, using the F1 score as the objective metric. The codes that implement the neural network are available at https://github.com/BHSAI/DNN-liverTox.

#### Random Forest

To evaluate the performance of the deep-learning method in relation to other methods, we also built RF classifiers. We constructed the RF models using Scikit-learn^6^. We optimized the hyperparameters (i.e., the number of trees, minimum impurity decrease; see Supplementary Table S3 for final values) by a randomized search technique with cross validation, using the F1 score as the objective metric.

#### Support Vector Machine

We also built SVM classifiers, using Scikit-learn (See footnote 6). We optimized the hyperparameters (i.e., the kernel used, value of locality parameter Gamma, and value of regularization parameter C; see Supplementary Table S3 for final values) by a randomized search technique with cross validation, using the F1 score as the objective metric.

## Results

### Performance of Four Machine-Learning Algorithms Using Cross Validation

**FIGURE 2:**
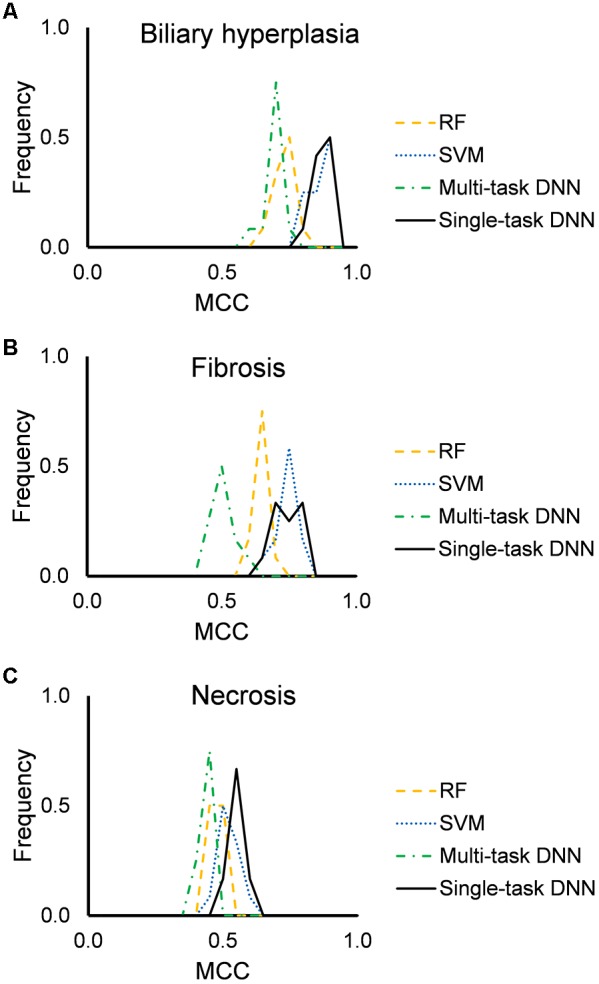
Performance of RF, SVM, single-task DNN, and multi-task DNN models based on cross validation studies. **(A–C)** results for biliary hyperplasia, fibrosis and necrosis, respectively.

Figure 2 shows the distribution of mean MCCs of the four machine-learning algorithms across the 12 feature sets. All algorithms achieved high MCC scores across these feature sets. Single-task DNN and SVM outperformed RF and multi-task DNN for the three endpoints. Tables 3, 4 show the performances on different endpoints for single-task DNN and SVM using different feature sets (the results for RF and multi-task DNN are shown in Supplementary Tables S4, S5). The single-task DNN and SVM perform similarly in these studies.

**Table 3:** Matthews correlation coefficients (MCCs) of single-task DNN models on cross validation data.

Feature set	Size	Biliary hyperplasia		Fibrosis		Necrosis	
				
		*Mean*	*SD*	*Mean*	*SD*	*Mean*	*SD*
**Gene-level feature sets**							
*Toxicity Module (L1000)*	154	0.85	0.09	0.77	0.15	0.53	0.07
*PTGS (core)*	199	0.82	0.11	0.73	0.12	0.51	0.06
*A200*	200	0.83	0.09	0.66	0.12	0.51	0.07
*A600*	600	0.87	0.08	0.75	0.18	0.55	0.08
*L1000*	978	0.87	0.06	0.72	0.18	**0.56**	0.10
*A1200*	1,200	0.85	0.05	0.77	0.13	0.55	0.09
*Toxicity Module Gene*	1,312	**0.89**	0.07	**0.78**	0.13	0.55	0.09
*PTGS (all)*	1,331	0.85	0.08	0.77	0.17	0.54	0.09
**Pathway-level feature sets**							
*MSigDB (hallmark)*	50	0.75	0.13	0.67	0.14	0.49	0.09
*Toxicity Module*	89	0.81	0.09	0.65	0.17	0.48	0.10
*MSigDB (C2) L1000*	1,220	0.83	0.09	0.67	0.21	0.53	0.08
*MSigDB (C2)*	1,329	0.82	0.08	0.68	0.16	0.51	0.07


*Bold indicates the greatest mean MCC value for each injury phenotype.*

**Table 4:** MCCs of SVM models on cross validation data.

Feature set	Size	Biliary hyperplasia		Fibrosis		Necrosis	
				
		*Mean*	*SD*	*Mean*	*SD*	*Mean*	*SD*
**Gene-level feature sets**							
*Toxicity Module (L1000)*	154	0.87	0.09	0.66	0.14	0.48	0.08
*PTGS (core)*	199	0.80	0.10	0.64	0.18	0.48	0.07
*A200*	200	0.79	0.07	0.70	0.12	0.55	0.09
*A600*	600	0.88	0.07	0.73	0.19	0.52	0.09
*L1000*	978	0.85	0.10	0.71	0.27	0.54	0.09
*A1200*	1,200	0.86	0.07	**0.80**	0.13	**0.57**	0.07
*Toxicity Module Gene*	1,312	**0.89**	0.07	0.75	0.18	0.51	0.07
*PTGS (all)*	1,331	0.84	0.06	0.73	0.15	0.49	0.08
**Pathway-level feature sets**							
*MSigDB (hallmark)*	50	0.79	0.13	0.71	0.17	0.44	0.10
*Toxicity Module*	89	0.83	0.10	0.71	0.18	0.46	0.05
*MSigDB (C2) L1000*	1,220	0.87	0.09	0.73	0.17	0.47	0.06
*MSigDB (C2)*	1,329	0.84	0.06	0.75	0.15	0.46	0.06


*Bold indicates the greatest mean MCC value for each injury phenotype.*

Based on the cross validation studies (Tables 3, 4), the best (i.e., highest average MCC) feature sets for single-task DNN were *Toxicity Module Gene* for the biliary hyperplasia and fibrosis endpoints and *L1000* for the necrosis endpoint; the best feature sets of SVM were *Toxicity Module Gene* for the biliary hyperplasia endpoint and *A1200* for the fibrosis and necrosis endpoints.

## Single-Task Dnn Had More Consistent Performance Than Svm for Data Not Seen in Training

Because SVM and single-task DNN models performed similarly in the cross validation studies, we further compared their performance on the Ippolito et al. data (see MATERIALS AND METHODS, Transcriptomic Data). We used this data because it was the largest external dataset we could find and contained more balanced endpoints annotation than all other external validation sets. To make an objective estimation of the performance, we removed the training samples related to the four chemicals occurring in the training dataset used to build the models (see MATERIALS AND METHODS, Transcriptomic Data). We compared the performance of single-task DNN and SVM algorithm using the models built on the best feature sets and found that single-task DNN outperformed SVM (Table 5). This result indicate that DNN models potentially exhibit better predictive ability for data not seen in training. Based on the above results, we selected the single-task DNN models built based on the *Toxicity Module Gene* feature set (for biliary hyperplasia and fibrosis) and *L1000* (for necrosis) for further studies.

**Table 5:** Performance of single-task DNN and SVM models using Ippolito et al. data.

	Biliary hyperplasia		Fibrosis		Necrosis	
			
	Best feature set	MCC	Best feature set	MCC	Best feature set	MCC
Single-task DNN	*Toxicity Module Gene*	0.76	*Toxicity Module Gene*	0.90	*L1000*	0.49
SVM	*Toxicity Module Gene*	0.67	*A1200*	0.79	*A1200*	0.36


### Specificity of Single-Task DNN Models

**FIGURE 3:**
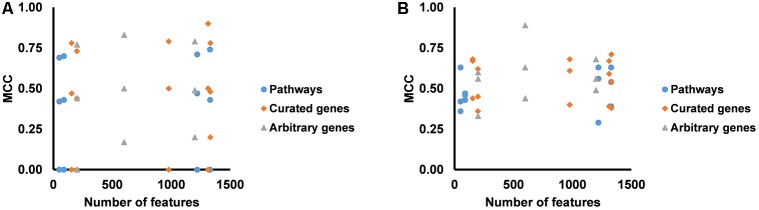
**(A)** MCCs for predicting presence of one endpoint and absence of two endpoints for all feature sets. **(B)** MCCs for predicting presence of two endpoints and absence of one endpoint for all feature sets.

We partitioned the training samples into different categories by the occurrence of the liver injury endpoints, i.e., three where only one endpoint was present (single-endpoint), three where exactly two endpoints were present (double-endpoint), and one where all three endpoints were present (Table 6, columns 1–3). We investigated the specificity of DNN models by evaluating their performance to predict single and multiple injury endpoints. Table 6 shows multiple metrics for the two best feature sets which had highest mean MCCs for the three endpoints. The results for the other feature sets are shown in Supplementary Table S6. We calculated these metrics using model predictions on all 10 validation sets. Figure 3 shows the MCCs of all 12 feature sets for single- and double-endpoint categories. In cross validation, most feature sets showed comparable levels of performance, exhibiting relatively high MCCs for most categories. All zero MCCs came from the category where only fibrosis occurred. This category had 2,809 negative samples, but only 5 positive samples. The extremely small size of the minor class, which only supports sparse sampling in the data space, may underlie the poor performance in this category. Overall, these results suggest that the single-task DNN models were specific to the injury endpoints.

**Table 6:** Metrics for three gene-level feature sets on combined cross validation data.

Biliary hyperplasia	Fibrosis	Necrosis	MCC	TP	TN	FN	FP	Sen	Spc	PPV	NPV	BAc	F1
**L1000**													
1	0	0	0.79	31	2,767	10	6	0.76	1.00	0.84	1.00	0.88	0.79
0	1	0	0.00	0	2,806	5	3	0.00	1.00	0.00	1.00	0.50	0.00
0	0	1	0.50	154	2,390	204	66	0.43	0.97	0.70	0.92	0.70	0.53
1	1	0	0.40	5	2,793	12	4	0.29	1.00	0.56	1.00	0.65	0.38
1	0	1	0.61	21	2,767	12	14	0.64	0.99	0.60	1.00	0.82	0.62
0	1	1	0.68	5	2,804	4	1	0.56	1.00	0.83	1.00	0.78	0.67
1	1	1	0.63	13	2,785	3	13	0.81	1.00	0.50	1.00	0.90	0.62
**Toxicity Module Gene**													
1	0	0	0.90	34	2,772	7	1	0.83	1.00	0.97	1.00	0.91	0.89
0	1	0	0.00	0	2,805	5	4	0.00	1.00	0.00	1.00	0.50	0.00
0	0	1	0.50	156	2,385	202	71	0.44	0.97	0.69	0.92	0.70	0.53
1	1	0	0.39	6	2,789	11	8	0.35	1.00	0.43	1.00	0.68	0.39
1	0	1	0.59	20	2,767	13	14	0.61	0.99	0.59	1.00	0.80	0.60
0	1	1	0.67	6	2,802	3	3	0.67	1.00	0.67	1.00	0.83	0.67
1	1	1	0.57	10	2,789	6	9	0.63	1.00	0.53	1.00	0.81	0.57


*BAc, Balanced Accuracy [ = ½(Sen + Spc)]; F1, F1 score; FN, false negatives; FP, false positives; MCC, Matthews correlation coefficient; NPV, negative predictive value [ = TN/(TN + FN)]; PPV, positive predictive value [ = TP/(TP + FP)]; Sen, sensitivity [ = TP/(TP + FN)]; Spc, specificity [ = TN/(FP + TN)]; TN, true negatives; TP, true positives.*

### External Validation Using Independent Data

**FIGURE 4:**
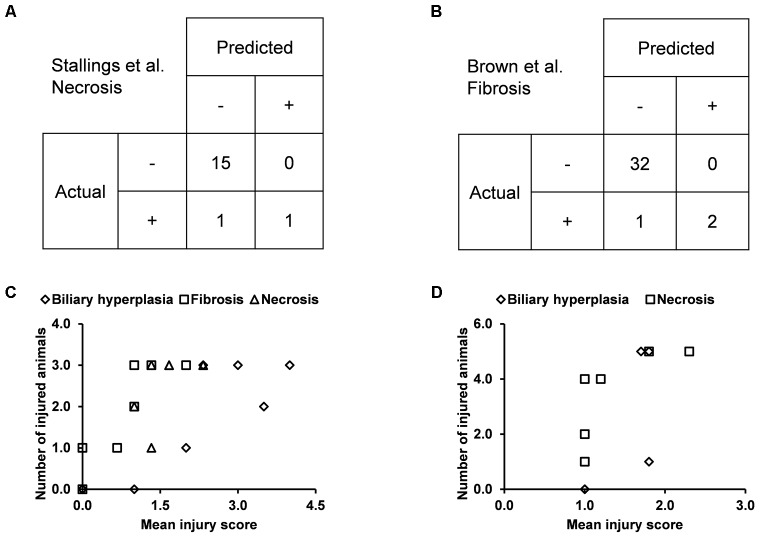
Performance of the single-task DNN model on four external testing sets. Contingency tables of prediction for data from **(A)** Stallings et al. and **(B)** Brown et al. **(C)** and **(D)** Correlation between measures of experimentally observed injury and model-predicted injury for data from **(C)** Sutherland et al., and **(D)** Slopianka et al. Each data point represents an exposure condition (a combination of treatment duration and dose). The experimental level of injury is given by the mean injury score based on histopathology evaluation, while the predicted level of injury is given by the number of positive samples.

We built the final single-task DNN models for the three endpoints and all training data using their corresponding best feature sets (i.e., *Toxicity Module Gene* for biliary hyperplasia and fibrosis; *L1000* for necrosis), and assessed the performance of these models on the other five external validation data. These datasets represented physiological and chemical perturbations independent of the training data (see MATERIALS AND METHODS, Transcriptomic Data). A comparison of the predictions with the injury annotations of Stallings et al. and Brown et al. (contingency matrices in Figure 4A,B, respectively) showed recall rates of 0.50 and 0.67, precision rates of 1.00 and 1.00, and F1 scores of 0.67 and 0.80, respectively, for the two datasets. For Eun et al., the contingency table was degenerative because there were no positive samples. Table 7 shows that the model correctly predicted almost all samples (4 erroneous predictions out of 807) for the three endpoints.

**Table 7:** Performance of single-task DNN in predicting Eun et al. data.

	Biliary hyperplasia	Fibrosis	Necrosis
Total	269	269	269
Correct	265	269	269
Missed	4	0	0
Accuracy (%)	98.5	100.0	100.0


Because the two other external datasets of Sutherland et al. and Slopianka et al. did not provide any endpoint injury annotations, we could not directly evaluate our predictions through standard contingency tables. However, these studies did provide average injury scores for multiple time points and/or doses. In addition, our predictions allowed us to derive the number of injured animals–another measure of the injury level–for the endpoints at these exposure conditions. Hence, if our predictions are accurate, the two measures should be positively correlated. Consistent with this expectation, the Spearman’s correlation coefficients (rho-values) were 0.72 (*p* = 7.8 × 10^-5^) for the dataset of Sutherland et al. (2018) and 0.73 (*p* = 0.006) for the dataset of Slopianka et al. (2017) (Figure 4C,D, respectively).

### Label-Shuffled Models Show No Predictive Power

We performed 10 random shufflings to construct 10 label-shuffled *Y-Shuffle* sets for *Toxicity Module Gene* and *L1000* (see MATERIALS AND METHODS, Feature Selection). Table 8 shows that these models performed poorly in both cross validation and for the external dataset of Ippolito et al. No meaningful model could be built from the label-shuffled dataset.

**Table 8:** Performance of Y-shuffled DNN models.

	Biliary hyperplasia		Fibrosis		Necrosis	
			
	Cross validation	Ippolito et al.	Cross validation	Ippolito et al.	Cross validation	Ippolito et al.
F1	0.03	0.01	0.02	0.00	0.14	0.16
Standard deviation	0.03	0.03	0.04	0.00	0.03	0.13


### Models With Arbitrary Gene Sets Show Poor Performance in External Validation

We also tested the performance of arbitrarily selected gene sets in predicting outcomes in the datasets of Stallings et al. and Brown et al. We used these datasets because they used treatment methods that differed from those used in the training data, and because the predictions could be directly compared with experimental results. The low F1 scores on the two datasets (Table 9) indicate that arbitrary gene features showed poor generalization performance.

**Table 9:** Mean F1 scores of arbitrary gene sets in predicting external testing sets.

Feature set	Brown et al.	Stallings et al.
		
	Fibrosis	Necrosis
*A200*	0.07	0.00
*A600*	0.05	0.00
*A1200*	0.29	0.07


## Discussion

### DNN Accurately Predict Liver Toxicity Endpoints From Transcriptomic Responses

Our single-task DNN model achieved high performance scores in cross validation using various feature selection methods (Table 3 and Figure 2). Our aim was to develop a model that learns signals associated not with specific chemical-exposure conditions but with the endpoints. The high MCCs for the three endpoints and various patterns of present/absent endpoints (Table 6 and Figure 3) indicate that our DNN models are endpoint-specific. We further validated our single-task DNN model with data from six additional independent experiments, most of which induced the target endpoints by treatments different from those used in the training data. The power of the single-task DNN model was highlighted by the fact that it performed satisfactorily across these datasets (Figure 3, 4 and Table 7). In summary, the single-task DNN model provided robust predictions for the intended injury phenotype.

To ascertain that the signals learned by our model was not spurious, we also applied y-randomization (Rucker et al., 2007) to the best feature sets for the three endpoints. In this method, the performance of the original model is compared to that of a model trained with the same original input variables and model-building procedure but with the output variable randomly shuffled. The underlying rationale is that a useful model should describe the given data better than by chance alone (Rucker et al., 2007); i.e., the model based on original data should outperform a model based on randomized data. We found that randomizing the class labels generated a model with no predictive ability (Table 8). All together, these results suggest that the DNN model captured true signals of the target endpoints.

### DNN Provides Robust Predictions for the Three Endpoints

Although DNNs have been extensively adopted in various fields of study in recent years, they are far from general panaceas. On many tasks, they perform no better than other methods. For example, we recently showed that the overall performance level of a DNN is quite similar to that of a variant of the nearest neighbor classifier (arguably the simplest machine-learning method) (Liu et al., 2018). Therefore, careful comparisons of DNNs with other machine-learning methods is important for identifying the strengths and weaknesses of this modeling approach. Here, we specifically chose RF and SVM models as a reference because, ever since their introduction at the turn of the century (Cortes and Vapnik, 1995; Breiman, 2001), they have proven successful in many fields, including the biomedical sciences (Denisko and Hoffman, 2018) and are strong competitors to DNN models in many areas. To ensure a fair comparison, we built RF and SVM models following the same procedure as that for constructing DNNs: searching the hyperparameter space to identify the best parameter settings, and then using these settings to build 10 models with 10-fold cross validation. Single-task DNN and SVM models showed comparable performance in cross validation studies (Tables 3, 4 and Figure 2), whereas, DNN models showed better performance when evaluated in the independent testing data (Table 5).

Comparing the performance of single-task DNN and SVM models using the external validation data also showed that single-task DNN models were less sensitive to the selection of feature set than SVM models for fibrosis (Supplementary Figure S1). SVM had mean MCCs of less than 0.05 (0.00, 0.15, 0.04, and 0.05) for feature set *PTGS core, A200, A600*, and *PTGS (all)*, while the corresponding MCC values for single-task DNN were 0.36, 0.60, 0.58, and 0.82, respectively. Furthermore, single-task DNN outperformed SVM in 27 out of 36 cases (Supplementary Figure S1).

Overall, DNN achieved consistent robust performance, which indicates that high-throughput *in vivo* toxicological expression data deposited in TG-GATEs and DrugMatrix contain rich information for predicting of these endpoints and DNN is a powerful method to extracting such information.

### Feature Selection and Model Performance

The curated feature sets can be classified into two types according to how they were selected. In gene-level features, *Toxicity Module Gene, PTGS (all)* and *PTGS (core)* are generated by data mining techniques to isolate the features associated with liver toxicity. In contrast, *L1000* is a reduced representation of genes sufficient to predict cell-wide gene expression patterns. In pathway level features, *Toxicity Module* features are generated by their statistical correlation with liver toxicity, while *MSigDB (C2)* and *MSigDB (C2) L1000* represent all known canonical pathways. The two types of feature sets shared only a small fraction of genes: the Jaccard index, an index of the similarity between two sets, was 0.11 for *L1000* and *PTGS (all)* and 0.08 for *MSigDB (C2)* and *Toxicity Module*. This finding indicates that the two types of feature sets are largely independent of each other. The comparable performance for the two types of features (Table 3) suggests that in constructing DNN model, providing input variables with enough diversity is more important than selecting the toxicity-specific features in advance. This notion is consistent with the ability of DNNs to automatically discover the relevant representations from the input features (LeCun et al., 2015).

In cross validation arbitrary gene sets did exhibit relatively high predictive power in the training data. For example, the mean MCCs for *A1200* the three endpoints are comparable to the best curated feature sets (Table 3). However, arbitrary gene sets performed poorly on external testing sets (Table 9). In contrast, the F1 scores for the best curated feature sets on the two external testing sets were 0.80 and 0.67 (Figure 4A,B), which were much higher than the corresponding scores of the models with arbitrary gene sets. The poor generalization of the arbitrary gene-set - based models indicated that they did not learn true signals associated with the endpoints as well as did the curated feature-set - based models.

For all three endpoints, the feature set *Toxicity Module Gene (L1000)* showed consistent performance in both cross validation and external validation for both single-task DNNs and SVMs (Tables 3, 4 and Supplementary Figure S1). This feature set contained only 154 features, but performed better than the other feature sets with similar number of features [*A200* and *PTGS (core)*] in the external Ippolito et al. data. In cross validation, small feature sets such as *Toxicity Module* and *MSigDB (Hallmark)* had in general lower mean performance than the best feature sets consisting of about 1,000 features. However, it is unlikely that the lower average performance for the two feature sets can be attributed to its small size, because as discussed above, the *Toxicity Module Gene (L1000)* feature set, which contained only 154 features, achieved F1 scores that were comparable to those of the best feature set. The correlation between feature set size and performance was rather low (*r^2^* = 0.02, *p* = 0.43; Table 3), indicating that the relationship between feature selection and model performance is not straightforward.

In summary, our findings show that gene- and pathway-level feature sets with diverse functional information perform on par with toxicity-specific feature sets; and single-task DNN have potential better performance better than SVM and RF; and carefully selection of features results in better performance than arbitrary selection in data not seen during training. Importantly, our DNNs exhibit good generalization of phenotype prediction in independent external testing datasets.

## Author Contributions

HW, RL, and AW conceived and designed the experiments. HW, RL, and PS developed the model and analyzed the data. HW, RL, PS, and AW contributed to the writing of the manuscript.

## Conflict of Interest Statement

The authors declare that the research was conducted in the absence of any commercial or financial relationships that could be construed as a potential conflict of interest.

## Footnotes

1 https://ntp.niehs.nih.gov/drugmatrix/index.html

2 http://toxico.nibiohn.go.jp

3 http://software.broadinstitute.org/gsea/msigdb/index.jsp

4 https://keras.io/

5 https://www.tensorflow.org/

6 http://scikit-learn.org/stable/index.html
