# Supplementary material for: Deep Neural Network Models for Predicting Chemically Induced Liver Toxicity Endpoints From Transcriptomic Responses
Source: Front Pharmacol. 2019 Feb 5;10:42. doi: 10.3389/fphar.2019.00042 (PMC6370634; doi:10.3389/fphar.2019.00042)
Supplement: Supplementary file 1 [file Data_Sheet_1.zip › ms-Table S4-S5.docx]

**Table S4: MCCs of RF models on cross validation data**

| **Feature set** | **Size** | **Biliary hyperplasia** | | **Fibrosis** | | **Necrosis** | |
| --- | --- | --- | --- | --- | --- | --- | --- |
|  |  | **Mean** | **SD** | **Mean** | **SD** | **Mean** | **SD** |
| **Gene-level feature sets** | | | | | | | |
| *Toxicity Module (L1000)* | 154 | 0.74 | 0.13 | 0.61 | 0.18 | 0.46 | 0.07 |
| *PTGS (core)* | 199 | 0.63 | 0.11 | 0.58 | 0.18 | 0.44 | 0.07 |
| *A200* | 200 | 0.69 | 0.11 | 0.59 | 0.17 | 0.43 | 0.06 |
| *A600* | 600 | 0.72 | 0.12 | 0.63 | 0.13 | 0.45 | 0.07 |
| *L1000* | 978 | 0.75 | 0.10 | 0.64 | 0.19 | 0.47 | 0.10 |
| *A1200* | 1,200 | 0.74 | 0.09 | 0.61 | 0.18 | 0.44 | 0.07 |
| *Toxicity Module Gene* | 1,312 | 0.77 | 0.10 | 0.68 | 0.14 | 0.48 | 0.08 |
| *PTGS (all)* | 1,331 | 0.71 | 0.11 | 0.65 | 0.20 | 0.48 | 0.09 |
| **Pathway-level feature sets** | | | | | | | |
| *MSigDB (hallmark)* | 50 | 0.66 | 0.09 | 0.62 | 0.22 | 0.41 | 0.07 |
| *Toxicity Module* | 89 | 0.68 | 0.11 | 0.61 | 0.19 | 0.45 | 0.09 |
| *MSigDB (C2) L1000* | 1,220 | 0.67 | 0.09 | 0.63 | 0.14 | 0.43 | 0.06 |
| *MSigDB (C2)* | 1,329 | 0.71 | 0.10 | 0.64 | 0.16 | 0.44 | 0.08 |

**Table S5: MCCs of multi-task DNN models on cross validation data**

| **Feature set** | **Size** | **Biliary hyperplasia** | | **Fibrosis** | | **Necrosis** | |
| --- | --- | --- | --- | --- | --- | --- | --- |
|  |  | **Mean** | **SD** | **Mean** | **SD** | **Mean** | **SD** |
| **Gene-level feature sets** | | | | | | | |
| *Toxicity Module (L1000)* | 154 | 0.68 | 0.07 | 0.49 | 0.09 | 0.42 | 0.09 |
| *PTGS (core)* | 199 | 0.70 | 0.04 | 0.50 | 0.06 | 0.43 | 0.07 |
| *A200* | 200 | 0.68 | 0.08 | 0.47 | 0.05 | 0.40 | 0.09 |
| *A600* | 600 | 0.70 | 0.07 | 0.46 | 0.07 | 0.42 | 0.08 |
| *L1000* | 978 | 0.67 | 0.08 | 0.45 | 0.07 | 0.44 | 0.08 |
| *A1200* | 1,200 | 0.64 | 0.09 | 0.44 | 0.08 | 0.42 | 0.08 |
| *Toxicity Module Gene* | 1,312 | 0.66 | 0.06 | 0.44 | 0.05 | 0.43 | 0.08 |
| *PTGS (all)* | 1,331 | 0.70 | 0.07 | 0.47 | 0.10 | 0.44 | 0.06 |
| **Pathway-level feature sets** | | | | | | | |
| *MSigDB (hallmark)* | 50 | 0.58 | 0.08 | 0.49 | 0.07 | 0.39 | 0.05 |
| *Toxicity Module* | 89 | 0.70 | 0.10 | 0.55 | 0.04 | 0.43 | 0.06 |
| *MSigDB (C2) L1000* | 1,220 | 0.67 | 0.07 | 0.50 | 0.04 | 0.38 | 0.03 |
| *MSigDB (C2)* | 1,329 | 0.71 | 0.07 | 0.51 | 0.08 | 0.38 | 0.04 |
