# Supplementary material for: Deep Neural Network Models for Predicting Chemically Induced Liver Toxicity Endpoints From Transcriptomic Responses
Source: Front Pharmacol. 2019 Feb 5;10:42. doi: 10.3389/fphar.2019.00042 (PMC6370634; doi:10.3389/fphar.2019.00042)
Supplement: Supplementary file 1 [file Data_Sheet_1.zip › ms-Table S6.docx]

**Table S6: Metrics for two pathway-level feature sets on combined cross validation data**

Abbreviations: MCC, Matthews correlation coefficient; TP, true positives; TN, true negatives; FN, false negatives; FP, false

positives; Sen, sensitivity [= TP / (TP + FN)]; Spc, specificity [= TN / (FP + TN)]; PPV, positive predictive value [= TP / (TP + FP)];

NPV, negative predictive value [= TN / (TN + FN)]; BAc, Balanced Accuracy [= ½ (Sen + Spc)]; F1, F1 score.

| Biliary hyperplasia | Fibrosis | Necrosis | MCC | TP | | TN | | FN | | FP | | Sen | | Spc | | PPV | | NPV | | BAc | | F1 | |  |
| --- | --- | --- | --- | --- | --- | --- | --- | --- | --- | --- | --- | --- | --- | --- | --- | --- | --- | --- | --- | --- | --- | --- | --- | --- |
| ***Toxicity Module Gene (L1000)*** | | | | | | | | | | | | | | | | | | | | | | | |  |
| 1 | 0 | 0 | 0.78 | | 31 | | 2,766 | | 10 | | 7 | | 0.76 | | 1.00 | | 0.82 | | 1.00 | | 0.88 | | 0.78 | |
| 0 | 1 | 0 | 0.00 | | 0 | | 2,806 | | 5 | | 3 | | 0.00 | | 1.00 | | 0.00 | | 1.00 | | 0.50 | | 0.00 | |
| 0 | 0 | 1 | 0.47 | | 142 | | 2,390 | | 216 | | 66 | | 0.40 | | 0.97 | | 0.68 | | 0.92 | | 0.68 | | 0.50 | |
| 1 | 1 | 0 | 0.44 | | 6 | | 2,792 | | 11 | | 5 | | 0.35 | | 1.00 | | 0.55 | | 1.00 | | 0.68 | | 0.43 | |
| 1 | 0 | 1 | 0.68 | | 22 | | 2,772 | | 11 | | 9 | | 0.67 | | 1.00 | | 0.71 | | 1.00 | | 0.83 | | 0.69 | |
| 0 | 1 | 1 | 0.67 | | 6 | | 2,802 | | 3 | | 3 | | 0.67 | | 1.00 | | 0.67 | | 1.00 | | 0.83 | | 0.67 | |
| 1 | 1 | 1 | 0.60 | | 11 | | 2,788 | | 5 | | 10 | | 0.69 | | 1.00 | | 0.52 | | 1.00 | | 0.84 | | 0.59 | |
| ***PTGS (core)*** | | | | | | | | | | | | | | | | | | | | | | | |  |
| 1 | 0 | 0 | 0.73 | 31 | | 2,761 | | 10 | | 12 | | 0.76 | | 1.00 | | 0.72 | | 1.00 | | 0.88 | | 0.74 | |  |
| 0 | 1 | 0 | 0.00 | 0 | | 2,802 | | 5 | | 7 | | 0.00 | | 1.00 | | 0.00 | | 1.00 | | 0.50 | | 0.00 | |  |
| 0 | 0 | 1 | 0.44 | 126 | | 2,399 | | 232 | | 57 | | 0.35 | | 0.98 | | 0.69 | | 0.91 | | 0.66 | | 0.47 | |  |
| 1 | 1 | 0 | 0.36 | 5 | | 2,791 | | 12 | | 6 | | 0.29 | | 1.00 | | 0.45 | | 1.00 | | 0.65 | | 0.36 | |  |
| 1 | 0 | 1 | 0.62 | 20 | | 2,770 | | 13 | | 11 | | 0.61 | | 1.00 | | 0.65 | | 1.00 | | 0.80 | | 0.63 | |  |
| 0 | 1 | 1 | 0.45 | 3 | | 2,803 | | 6 | | 2 | | 0.33 | | 1.00 | | 0.60 | | 1.00 | | 0.67 | | 0.43 | |  |
| 1 | 1 | 1 | 0.61 | 11 | | 2,789 | | 5 | | 9 | | 0.69 | | 1.00 | | 0.55 | | 1.00 | | 0.84 | | 0.61 | |  |
| ***A200*** | | | | | | | | | | | | | | | | | | | | | | | |  |
| 1 | 0 | 0 | 0.77 | 32 | | 2,763 | | 9 | | 10 | | 0.78 | | 1.00 | | 0.76 | | 1.00 | | 0.89 | | 0.77 | |  |
| 0 | 1 | 0 | 0.00 | 0 | | 2,802 | | 5 | | 7 | | 0.00 | | 1.00 | | 0.00 | | 1.00 | | 0.50 | | 0.00 | |  |
| 0 | 0 | 1 | 0.44 | 123 | | 2,404 | | 235 | | 52 | | 0.34 | | 0.98 | | 0.70 | | 0.91 | | 0.66 | | 0.46 | |  |
| 1 | 1 | 0 | 0.33 | 5 | | 2,789 | | 12 | | 8 | | 0.29 | | 1.00 | | 0.38 | | 1.00 | | 0.65 | | 0.33 | |  |
| 1 | 0 | 1 | 0.56 | 17 | | 2,771 | | 16 | | 10 | | 0.52 | | 1.00 | | 0.63 | | 0.99 | | 0.76 | | 0.57 | |  |
| 0 | 1 | 1 | 0.60 | 4 | | 2,804 | | 5 | | 1 | | 0.44 | | 1.00 | | 0.80 | | 1.00 | | 0.72 | | 0.57 | |  |
| 1 | 1 | 1 | 0.45 | 9 | | 2,782 | | 7 | | 16 | | 0.56 | | 0.99 | | 0.36 | | 1.00 | | 0.78 | | 0.44 | |  |
| ***A600*** | | | | | | | | | | | | | | | | | | | | | | | |  |
| 1 | 0 | 0 | 0.83 | 33 | | 2,768 | | 8 | | 5 | | 0.80 | | 1.00 | | 0.87 | | 1.00 | | 0.90 | | 0.84 | |  |
| 0 | 1 | 0 | 0.17 | 1 | | 2,803 | | 4 | | 6 | | 0.20 | | 1.00 | | 0.14 | | 1.00 | | 0.60 | | 0.17 | |  |
| 0 | 0 | 1 | 0.50 | 152 | | 2,391 | | 206 | | 65 | | 0.42 | | 0.97 | | 0.70 | | 0.92 | | 0.70 | | 0.53 | |  |
| 1 | 1 | 0 | 0.44 | 7 | | 2,789 | | 10 | | 8 | | 0.41 | | 1.00 | | 0.47 | | 1.00 | | 0.70 | | 0.44 | |  |
| 1 | 0 | 1 | 0.63 | 22 | | 2,767 | | 11 | | 14 | | 0.67 | | 0.99 | | 0.61 | | 1.00 | | 0.83 | | 0.64 | |  |
| 0 | 1 | 1 | 0.89 | 8 | | 2,804 | | 1 | | 1 | | 0.89 | | 1.00 | | 0.89 | | 1.00 | | 0.94 | | 0.89 | |  |
| 1 | 1 | 1 | 0.73 | 13 | | 2,791 | | 3 | | 7 | | 0.81 | | 1.00 | | 0.65 | | 1.00 | | 0.90 | | 0.72 | |  |
| ***A1200*** | | | | | | | | | | | | | | | | | | | | | | | |  |
| 1 | 0 | 0 | 0.79 | 33 | | 2,764 | | 8 | | 9 | | 0.80 | | 1.00 | | 0.79 | | 1.00 | | 0.90 | | 0.80 | |  |
| 0 | 1 | 0 | 0.20 | 1 | | 2,805 | | 4 | | 4 | | 0.20 | | 1.00 | | 0.20 | | 1.00 | | 0.60 | | 0.20 | |  |
| 0 | 0 | 1 | 0.49 | 151 | | 2,388 | | 207 | | 68 | | 0.42 | | 0.97 | | 0.69 | | 0.92 | | 0.70 | | 0.52 | |  |
| 1 | 1 | 0 | 0.49 | 7 | | 2,792 | | 10 | | 5 | | 0.41 | | 1.00 | | 0.58 | | 1.00 | | 0.70 | | 0.48 | |  |
| 1 | 0 | 1 | 0.56 | 18 | | 2,768 | | 15 | | 13 | | 0.55 | | 1.00 | | 0.58 | | 0.99 | | 0.77 | | 0.56 | |  |
| 0 | 1 | 1 | 0.68 | 5 | | 2,804 | | 4 | | 1 | | 0.56 | | 1.00 | | 0.83 | | 1.00 | | 0.78 | | 0.67 | |  |
| 1 | 1 | 1 | 0.60 | 12 | | 2,785 | | 4 | | 13 | | 0.75 | | 1.00 | | 0.48 | | 1.00 | | 0.87 | | 0.59 | |  |
| ***PTGS (all)*** | | | | | | | | | | | | | | | | | | | | | | | |  |
| 1 | 0 | 0 | 0.78 | 31 | | 2,766 | | 10 | | 7 | | 0.76 | | 1.00 | | 0.82 | | 1.00 | | 0.88 | | 0.78 | |  |
| 0 | 1 | 0 | 0.20 | 1 | | 2,805 | | 4 | | 4 | | 0.20 | | 1.00 | | 0.20 | | 1.00 | | 0.60 | | 0.20 | |  |
| 0 | 0 | 1 | 0.48 | 159 | | 2,368 | | 199 | | 88 | | 0.44 | | 0.96 | | 0.64 | | 0.92 | | 0.70 | | 0.53 | |  |
| 1 | 1 | 0 | 0.38 | 5 | | 2,792 | | 12 | | 5 | | 0.29 | | 1.00 | | 0.50 | | 1.00 | | 0.65 | | 0.37 | |  |
| 1 | 0 | 1 | 0.54 | 17 | | 2,769 | | 16 | | 12 | | 0.52 | | 1.00 | | 0.59 | | 0.99 | | 0.76 | | 0.55 | |  |
| 0 | 1 | 1 | 0.71 | 6 | | 2,803 | | 3 | | 2 | | 0.67 | | 1.00 | | 0.75 | | 1.00 | | 0.83 | | 0.71 | |  |
| 1 | 1 | 1 | 0.59 | 12 | | 2,784 | | 4 | | 14 | | 0.75 | | 0.99 | | 0.46 | | 1.00 | | 0.87 | | 0.57 | |  |
| ***MSigDB (hallmark)*** | | | | | | | | | | | | | | | | | | | | | | | |  |
| 1 | 0 | 0 | 0.69 | 28 | | 2,761 | | 13 | | 12 | | 0.68 | | 1.00 | | 0.70 | | 1.00 | | 0.84 | | 0.69 | |  |
| 0 | 1 | 0 | 0.00 | 0 | | 2,805 | | 5 | | 4 | | 0.00 | | 1.00 | | 0.00 | | 1.00 | | 0.50 | | 0.00 | |  |
| 0 | 0 | 1 | 0.42 | 140 | | 2,359 | | 218 | | 97 | | 0.39 | | 0.96 | | 0.59 | | 0.92 | | 0.68 | | 0.47 | |  |
| 1 | 1 | 0 | 0.36 | 3 | | 2,796 | | 14 | | 1 | | 0.18 | | 1.00 | | 0.75 | | 1.00 | | 0.59 | | 0.29 | |  |
| 1 | 0 | 1 | 0.42 | 14 | | 2,763 | | 19 | | 18 | | 0.42 | | 0.99 | | 0.44 | | 0.99 | | 0.71 | | 0.43 | |  |
| 0 | 1 | 1 | 0.63 | 5 | | 2,803 | | 4 | | 2 | | 0.56 | | 1.00 | | 0.71 | | 1.00 | | 0.78 | | 0.63 | |  |
| 1 | 1 | 1 | 0.59 | 12 | | 2,784 | | 4 | | 14 | | 0.75 | | 0.99 | | 0.46 | | 1.00 | | 0.87 | | 0.57 | |  |
| ***Toxicity Module*** | | | | | | | | | | | | | | | | | | | | | | | |  |
| 1 | 0 | 0 | 0.70 | 27 | | 2,764 | | 14 | | 9 | | 0.66 | | 1.00 | | 0.75 | | 0.99 | | 0.83 | | 0.70 | |  |
| 0 | 1 | 0 | 0.00 | 0 | | 2,807 | | 5 | | 2 | | 0.00 | | 1.00 | | 0.00 | | 1.00 | | 0.50 | | 0.00 | |  |
| 0 | 0 | 1 | 0.43 | 148 | | 2,346 | | 210 | | 110 | | 0.41 | | 0.96 | | 0.57 | | 0.92 | | 0.68 | | 0.48 | |  |
| 1 | 1 | 0 | 0.43 | 5 | | 2,794 | | 12 | | 3 | | 0.29 | | 1.00 | | 0.63 | | 1.00 | | 0.65 | | 0.40 | |  |
| 1 | 0 | 1 | 0.46 | 15 | | 2,765 | | 18 | | 16 | | 0.45 | | 0.99 | | 0.48 | | 0.99 | | 0.72 | | 0.47 | |  |
| 0 | 1 | 1 | 0.47 | 4 | | 2,801 | | 5 | | 4 | | 0.44 | | 1.00 | | 0.50 | | 1.00 | | 0.72 | | 0.47 | |  |
| 1 | 1 | 1 | 0.59 | 12 | | 2,784 | | 4 | | 14 | | 0.75 | | 0.99 | | 0.46 | | 1.00 | | 0.87 | | 0.57 | |  |
| ***MSigDB (C2) L1000*** | | | | | | | | | | | | | | | | | | | | | | | |  |
| 1 | 0 | 0 | 0.71 | 31 | | 2,758 | | 10 | | 15 | | 0.76 | | 0.99 | | 0.67 | | 1.00 | | 0.88 | | 0.71 | |  |
| 0 | 1 | 0 | 0.00 | 0 | | 2,806 | | 5 | | 3 | | 0.00 | | 1.00 | | 0.00 | | 1.00 | | 0.50 | | 0.00 | |  |
| 0 | 0 | 1 | 0.47 | 131 | | 2,407 | | 227 | | 49 | | 0.37 | | 0.98 | | 0.73 | | 0.91 | | 0.67 | | 0.49 | |  |
| 1 | 1 | 0 | 0.29 | 4 | | 2,790 | | 13 | | 7 | | 0.24 | | 1.00 | | 0.36 | | 1.00 | | 0.62 | | 0.29 | |  |
| 1 | 0 | 1 | 0.56 | 17 | | 2,771 | | 16 | | 10 | | 0.52 | | 1.00 | | 0.63 | | 0.99 | | 0.76 | | 0.57 | |  |
| 0 | 1 | 1 | 0.63 | 6 | | 2,801 | | 3 | | 4 | | 0.67 | | 1.00 | | 0.60 | | 1.00 | | 0.83 | | 0.63 | |  |
| 1 | 1 | 1 | 0.53 | 10 | | 2,786 | | 6 | | 12 | | 0.63 | | 1.00 | | 0.45 | | 1.00 | | 0.81 | | 0.53 | |  |
| ***MSigDB (C2)*** | | | | | | | | | | | | | | | | | | | | | | | |  |
| 1 | 0 | 0 | 0.74 | 29 | | 2,765 | | 12 | | 8 | | 0.71 | | 1.00 | | 0.78 | | 1.00 | | 0.85 | | 0.74 | |  |
| 0 | 1 | 0 | 0.00 | 0 | | 2,808 | | 5 | | 1 | | 0.00 | | 1.00 | | 0.00 | | 1.00 | | 0.50 | | 0.00 | |  |
| 0 | 0 | 1 | 0.43 | 130 | | 2,382 | | 228 | | 74 | | 0.36 | | 0.97 | | 0.64 | | 0.91 | | 0.67 | | 0.46 | |  |
| 1 | 1 | 0 | 0.39 | 6 | | 2,789 | | 11 | | 8 | | 0.35 | | 1.00 | | 0.43 | | 1.00 | | 0.68 | | 0.39 | |  |
| 1 | 0 | 1 | 0.54 | 17 | | 2,768 | | 16 | | 13 | | 0.52 | | 1.00 | | 0.57 | | 0.99 | | 0.76 | | 0.54 | |  |
| 0 | 1 | 1 | 0.63 | 5 | | 2,803 | | 4 | | 2 | | 0.56 | | 1.00 | | 0.71 | | 1.00 | | 0.78 | | 0.63 | |  |
| 1 | 1 | 1 | 0.46 | 9 | | 2,783 | | 7 | | 15 | | 0.56 | | 0.99 | | 0.38 | | 1.00 | | 0.78 | | 0.45 | |  |
