# Supplementary material for: Deep Neural Network Models for Predicting Chemically Induced Liver Toxicity Endpoints From Transcriptomic Responses
Source: Front Pharmacol. 2019 Feb 5;10:42. doi: 10.3389/fphar.2019.00042 (PMC6370634; doi:10.3389/fphar.2019.00042)

Figure S1: Performance of SVM and single-task DNN on Ippolito et al. data. TMG-L1000: *Toxicity Module Gene (L1000)*; Hallmark: *MSigDB (hallmark)*; C2 L1000: *MSigDB (C2) L1000*; C2: *MSigDB (C2)*.

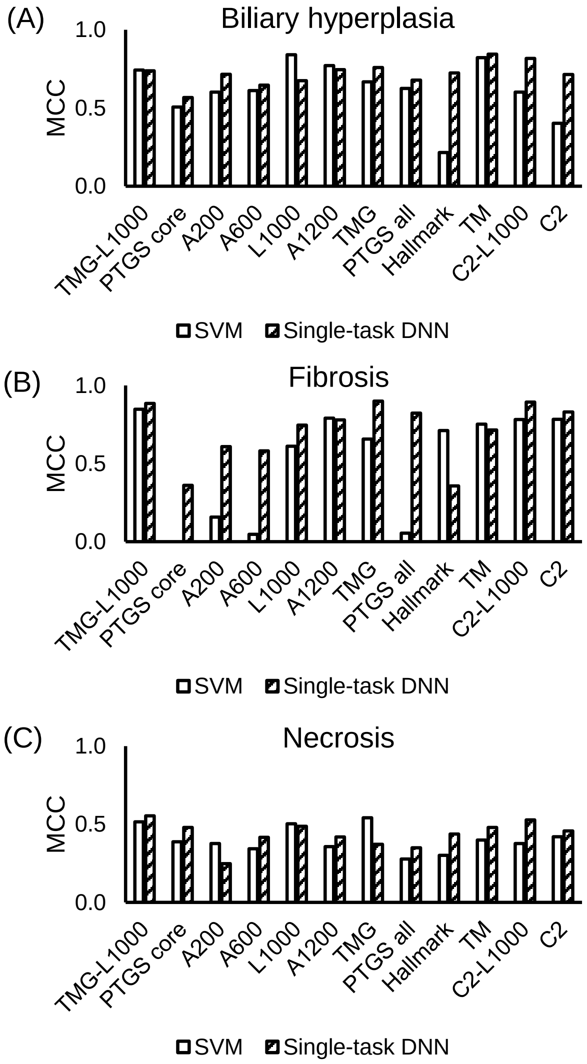

Supplement: Supplementary file 2 [file Data_Sheet_2.PDF]
